# The effects of lower limb ischaemic preconditioning: a systematic review

**DOI:** 10.3389/fphys.2023.1323310

**Published:** 2024-01-11

**Authors:** Chloe French, Dan Robbins, Marie Gernigon, Dan Gordon

**Affiliations:** ^1^ Cambridge Centre for Sport and Exercise Sciences (CCSES), Faculty of Science and Engineering, Anglia Ruskin University, Cambridge, United Kingdom; ^2^ CIAMS, Université Paris-Saclay, Orsay Cedex, France; ^3^ CIAMS, Université d’Orléans, Orléans, France; ^4^ Medical Technology Research Centre, Faculty of Health, Education, Medicine and Social Care, Anglia Ruskin University, Chelmsford, United Kingdom

**Keywords:** ischaemic preconditioning, blood flow restriction, lower limb, oxygenation, near infrared spectroscopy

## Abstract

Ischaemic preconditioning (IPC) involves the use of repeated occlusions and reperfusions of the peripheral muscle blood supply at a limb. This systematic literature review examines the typical responses in response to the method of application during an IPC applied at the lower limb. This review focuses on the physiological responses for VO_2max_, haemoglobin, metabolic and genetic responses to various IPC interventions. The literature search was performed using four databases and assessed using the PRISMA search strategy and COSMIN to assess the quality of the articles. Seventeen articles were included in the review, with a total of 237 participants. While there is variation in the method of application, the average occlusion pressure was 222 ± 34 mmHg, ranging from 170 to 300 mmHg typically for 3 or 4 occlusion cycles. The distribution of this pressure is influenced by cuff width, although 8 studies failed to report cuff width. The majority of studies applies IPC at the proximal thigh with 16/17 studies applying an occlusion below this location. The results highlighted the disparities and conflicting findings in response to various IPC methods. While there is some agreement in certain aspects of the IPC manoeuvre such as the location of the occlusion during lower limb IPC, there is a lack of consensus in the optimal protocol to elicit the desired responses. This offers the opportunity for future research to refine the protocols, associated responses, and mechanisms responsible for these changes during the application of IPC.

## 1 Introduction

Ischaemic preconditioning (IPC) involves repeated cycles of alternating occlusion and reperfusion, more recently adopted as a modality that precedes exercise (De Groot et al., 2010; Paradis-Deschênes et al., 2016). IPC originally stemmed from a clinical background as a protective mechanism for ischaemic-reperfusion injury with cardioprotective effects (Murry et al., 1986), with much of the earlier research performed in animal studies. IPC involves the application of an occlusion to a limb with protective effects in remote organs or tissues, resulting in an improved endothelial function and microcirculation (Kraemer et al., 2011; Gu et al., 2021). IPC can also promote local benefits, such as an improved peripheral vascular response (O’Brien and Jacobs, 2022). More recently, IPC has been applied as a non-invasive technique in a sporting context to improve performance and recovery (Sharma et al., 2015). The ergogenic effects across different exercise intensities and modalities, specifically include improvements in maximal oxygen uptake (VO_2max_) (De Groot et al., 2010), cycling time trials (Cocking et al., 2018), 5-km running time trial duration (Bailey et al., 2012) and maximal swimming time performance (Jean-St-Michel et al., 2011). To the contrary, other studies have identified no improvements in submaximal performance, cycling time trials or running economy (Clevidence et al., 2012; Kaur et al., 2017). Despite the conflicting findings, IPC is typically used to promote enhancements in performance-based outcomes, with investigations into vascular function and oxygenation. These components may contribute to the underlying mechanisms, physiology, and the overall cellular adaptation (De Groot et al., 2010).

The proposed mechanisms derive from neuronal, immune, and systemic mechanisms and pathways (Lang and Kim, 2022). However, it can be difficult to determine if there is an interconnection in the mechanisms, although there is a likely overlap. The use of near-infrared spectroscopy (NIRS) can be used to estimate the concentration of oxygenated and deoxygenated haemoglobin in the tissue (Barstow, 2019). IPC can improve vasodilation and oxygen delivery to the working muscles to improve performance, alongside improvements in the deoxygenation dynamics (Kido et al., 2015). However, the haemoglobin responses vary considerably for IPC (Paradis-Deschenes et al., 2017; Jeffries et al., 2019; Huang et al., 2020), highlighting the large variability in the existing research. The increase in blood flow following the reperfusion during the IPC manoeuvre results in an increase in shear stress in the endothelium (Gu et al., 2021). Shear stress acts as a stimulus for the release of nitric oxide (NO) from endothelium nitric oxide synthase (eNOS) (Lang and Kim, 2022), with repeated exposure resulting in an increase eNOS transcription and a greater bioavailability of NO (Hunt et al., 2013). Indeed, the increased NO production results in beneficial vasodilatory effects. The shear stress resulting from the arterial occlusion during repeated IPC interventions can act as a stimulus for arteriogenesis, which is the development of collateral blood vessels and arteries from existing arterioles (Schaper, 2009). This in turn can increase artery diameter and blood flow, enhancing the microvascular circulation and the process of vascular remodelling (Schaper and Scholz, 2003; Cooke and Meng, 2020). However, this process is unlikely to occur during the time frames of acute IPC.

Alternative mechanisms have been suggested such as an increased activation of the ATP-sensitive potassium channels (Riksen et al., 2004). An increase in substrate provision within the intracellular kinase pathways increases mitochondrial density resulting in the opening of ATP-sensitive potassium channels. This in turn reduces the permeability in the mitochondrial transition pore, creating a protective mechanism (Halestrap et al., 2007). The shielding effect created by the reduction in permeability has been suggested to formulate a window of protection imminently in the hours following the IPC, with a potential second delayed window after 24-h (Riksen et al., 2004) which could remain effective for a further 48–72-h post-application (Loukogeorgakis et al., 2005). This is an important consideration in the application of acute IPC to ensure the beneficial responses to IPC are not mistaken for the phases of protection.

The current evidence base highlights the disparities in the methods used for IPC, the physiological responses, and the mechanisms responsible. With the differences and inconsistencies in the methods and outcomes with no optimal approach defined, the purpose of this systematic review is to investigate the common methods of application and the typical responses associated with IPC. The primary physiological responses that will be investigated are VO_2max_, haemoglobin concentration, vascular, blood lactate and genetic responses to various IPC interventions. The aim is to provide a better understanding of the occlusion protocols at the lower limb and whether there is an optimal method required to elicit the desired responses.

## 2 Method

**FIGURE 1:**
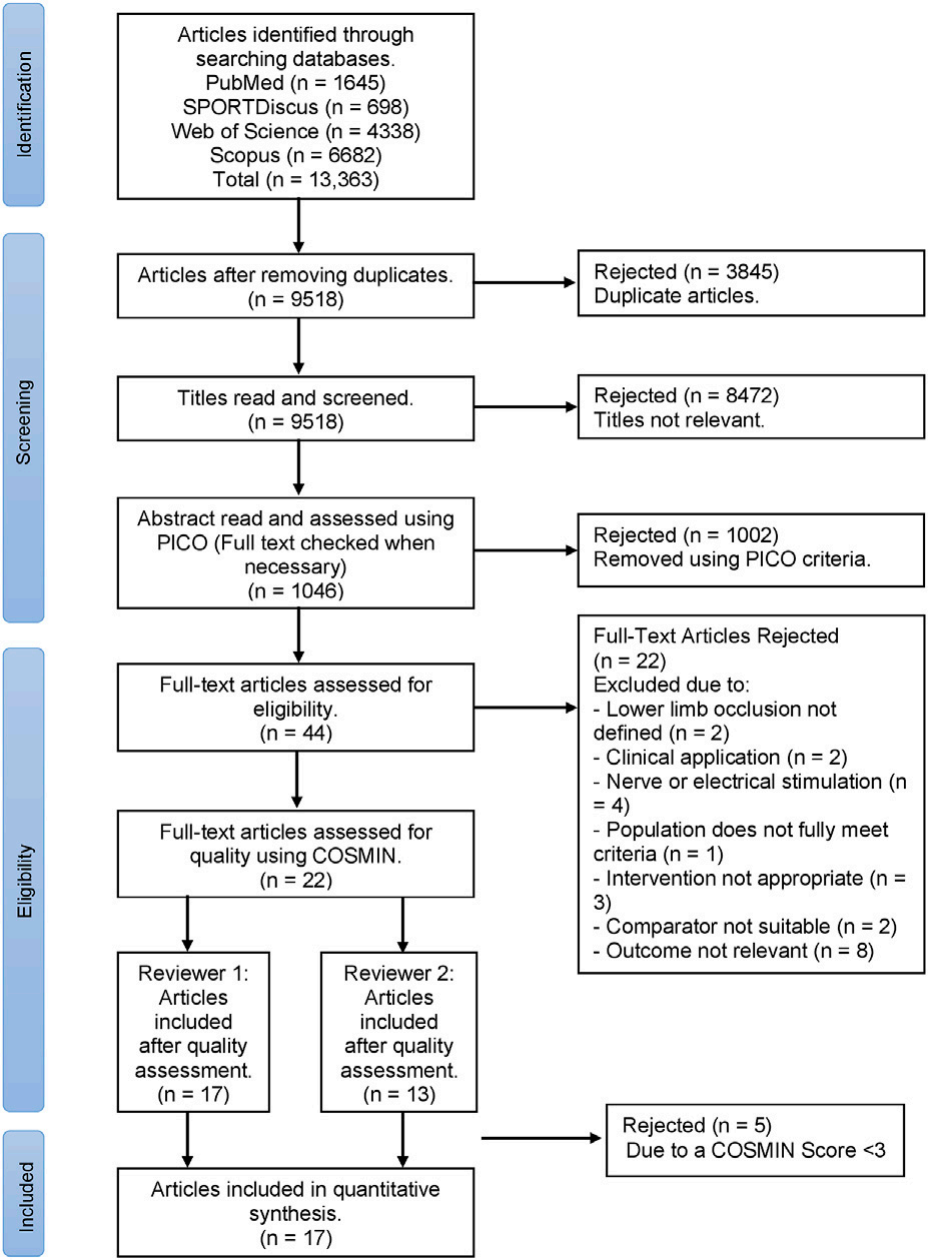
PRISMA flow diagram for the selection process of articles in this systematic review, where n = number of studies.

The systematic review was conducted using guidance from PRISMA (Preferred Reporting Items for Systematic Reviews and Meta-Analyses) (Moher et al., 2009) combined with Cochrane Handbook for Systematic Reviews (Higgins et al., 2019), as displayed in a flow diagram (Figure 1). The review has been registered with PROPSERO (CRD42023408123).

### 2.1 Search strategy

A literature search was conducted in September 2022, incorporating four databases including PubMed, SportDiscus (EBSCO), Scopus (Elsevier) and Web of Science (CLARIVATE). The search terms adopted were (“ischaemic preconditioning” OR “ischemic preconditioning” OR “blood flow training” OR “blood flow restriction” OR “blood flow occlusion” OR “occlusion therapy”) AND (Physiol* OR biolog* OR neurolog* OR metabolism OR oxygenation OR vascular). The results were exported into a reference management system (Mendeley, Elsevier, London, United Kingdom), assessed for duplicates and screened using Microsoft Excel V16.65 (Microsoft Corporation, Washington, United States).

### 2.2 Study selection

Abstracts were screened using a PICO-based criterion (population, intervention, comparison, outcome) as presented in Table 1.

**TABLE 1:** PICO criteria used in the article selection.

PICO	Criteria
*Population*	Human participants
	Males AND/OR Females
	18–60-years-old
	Healthy/Non-Clinical
*Intervention*	Method of IPC (e.g., occlusion pressure)
	Lower limb application with location defined (e.g., proximal thigh)
	Non-surgical interventions
	Can include local or remote application
*Comparator*	Comparison of intervention method used, OR Comparison to a control or sham group, OR Pre- vs post-intervention studies, OR Reliability/validity studies
*Outcome*	An indicator of change in a measure of IPC:
	**Physiological**—VO_2max_
	**Vascular**—e.g., Ankle Brachial Index (ABI), Flow Mediated Dilation (FMD), Pulse Wave Velocity (PWV)
	**Oxygenation**—e.g., O_2_Hb, HHb, THb, TSI.
	**Metabolic**—e.g., Lactate, PCr
	**Gene Expression**—e.g., VEGF, HIF1α, IGF1

### 2.3 Quality assessment

A quality assessment was carried out using Consensus-Based Standards for the Selection of Health Status Measurement Instruments (COSMIN) (Mokkink et al., 2018). Eighteen questions were assessed, with each section rated using the “worst score counts” approach. The inclusion threshold was defined as a COSMIN score ≥3 for a sufficient quality to be accepted into the review (Terwee et al., 2012). Two reviewers independently repeated the process to establish and improve the reproducibility of the review and reduce bias (Stoll et al., 2019).

### 2.4 Data extraction

The following data was extracted into Microsoft Excel V16.74 (Microsoft Corporation, Washington, United States) from each of the accepted articles: sample size, participant characteristics (population, age, sex), IPC protocol (limb, pressure, cycles), exercise protocol and any outcomes specified in the PICO criteria (Table 1).

### 2.5 Data analysis

Effect sizes were calculated using Cohens *d* (2013) effect size, which are classified as small: *d* = 0.2; medium: *d* = 0.5 and large: *d* = 0.8.

## 3 Results

### 3.1 Study selection and quality assessment

Figure 1 presents the PRISMA flow chart for the selection of articles presented in the systematic review. The COSMIN quality assessment identified 17 out of 22 articles as eligible to be included in the systematic review (Table 2).

**TABLE 2:** Overview of ischaemic preconditioning (IPC) studies accepted in the systematic review, where ↑ = increase, ↓ = decrease and NC = no change.

Study	Sample size	Population	Age (years)	IPC protocol	Type of exercise	Exercise protocol	Outcome
*Clevidence, Mowery and Kushnick (2012)*	M12	Healthy Male Competitive Amateur Level Cyclists	26.7 ± 8.6	Thigh (bilateral)—proximal	Cycling	Maximal graded cycle test: 100 W for 5-mins. 30 W⋅min^−1^ increments. Submaximal test: 30%, 50%, 70% MPO for 5-mins. 90% MPO to exhaustion	No significant difference in BLa or Glucose between IPC and control at each intensity
				220 mmHg			
				3 × 5-mins			
				Control: Cuff but no inflation			
				1 IPC and 1 control session, separated by 2–7 days			
				Cuff Width: NR (KAATSU Mini Master, Sato Sports Plaza Ltd., Tokyo, Japan)			
*Cruz et al. (2015)*	M12	Recreational Trained Male Cyclists	20–36	Thigh (bilateral)—proximal	Cycling	Incremental cycling test: 0.5 W⋅kg^−1^ increments every 3-mins. Tlim (time to exhaustion) test: 3-min cycling. 100% PPO to exhaustion	↑ VO_2peak_ by 2.9% in the IPC condition. No difference in BLa between IPC and control
				220 mmHg			
				4 × 5-mins			
				Control: 20 mmHg			
				1 IPC and 1 control session, separated by minimum 48-h (repeated for reliability)			
				Cuff: NR			
*Cocking et al. (2018)*	12	Cyclists	36.0 ± 7.0	Thigh (unilateral and bilateral)—proximal	Cycling	375-kJ time trial. Maximal Incremental Cycling Test: Start: 95 W. 35 W increments every 3-mins	↓ VO_2_ after traditional IPC (possibly beneficial). ↑ BLa after traditional IPC (possibly trivial). No additional benefit from 8x5-min protocol
				220 mmHg			
				Traditional: 4 × 5-mins. Larger: 8 × 5-mins			
				Sham: 20 mmHg			
				4 IPC and 1 sham session, separated by minimum 4 days			
				Cuff Width: 13.5 cm (brand NR)			
*De Groot et al. (2010)*	M12; F3	Healthy, Well-Trained Subjects	27.2 ± 5.6	Thigh (bilateral)—proximal	Cycling	Incremental maximal cycling test: Start: 50 W for 5-mins, 100 W for 4-mins, 150 W for 4-mins. 20 W⋅min^−1^ increments to exhaustion	↑ VO_2max_ after IPC. No difference in BLa following IPC or control
				220 mmHg			
				3 × 5-mins			
				Control: No restriction			
				1 IPC and 1 control session, separated by 7 days			
				Cuff: NR			
*Griffin et al. (2018)*	M12	Recreationally Active Males	30.0 ± 6.0	Thigh (bilateral)—proximal	Cycling	2x all-out cycling tests: 3-mins	↓ TSI during IPC occlusion. NC in TSI between conditions during the all-out test. No difference in VO_2peak_ or BLa in either condition
				220 mmHg			
				4 × 5-mins			
				Sham: 20 mmHg			
				1 IPC and 1 sham session, separated by 7 days			
				Cuff Width: 14.5 cm (Delfi Medical Innovations, Vancouver, Canada)			
*Huang et al. (2020)*	M14	Healthy Non-Athletic Males	26.0 ± 3.5	Thigh (bilateral)—anterior midline	Knee Extension	Isokinetic strength tests: 6 familiarisation trials, then 3 × 30°s^−1^, 3 × 150°⋅s^−1^, 3 × 270°s^−1^ Isokinetic endurance tests: 6 familiarisation trials, then 30 × 180◦s^−1^	↑ Resting THb following IPC. NC in THb following sham. NC in O_2_Hb, HHb and SaO_2_ after IPC or sham
				50 mmHg above SBP (∼170 mmHg)			
				4 × 5-mins			
				Sham: 10 mmHg			
				1 IPC and 1 sham session, separated by 7 days			
				Cuff Width: 14.2 cm (Spirit(R) P-106NXL+P-107ADT, Taiwan)			
*Jeffries et al. (2019)*	M20	Healthy Males	Overall: 21.0 ± 2.0 IPC: 22.0 ± 3.0 Sham: 21.0 ± 2.0	Thigh (bilateral)—proximal	Cycling	Maximal incremental cycling test: Start: 120 W. 35 W⋅min^−1^. Sub-maximal cycling at 70%, 80%, 90% of VT.	NC in VO_2max._ End BLa during VO_2max_ ↑ significantly, but NC in sub-maximal cycling. NC in THb at 70%, 80% or 90%. ↓ ΔHHb at 70% and 80% after IPC.
				220 mmHg			
				4 × 5-mins			
				Sham: 20 mmHg			
				7 consecutive days of IPC or sham			
				Cuff Width: 14.5 cm (Delfi Medical Innovations, Vancouver, Canada)			
*Kaur et al. (2017)*	M12; F6	Young and Middle-Aged Habitual Runners	27.0 ± 7.0	Thigh (bilateral)—proximal	Treadmill Running	2x incremental sub-maximal treadmill tests: 65%–85% VO_2max._ 3 × 5-min stages (7.2–14.5 km⋅hr^−1^)	NC in running economy. ↑ BLa in IPC and sham from baseline. NC between conditions
				220 mmHg			
				3 × 5-mins			
				Sham: 20 mmHg			
				1 IPC and 1 sham session, separated by at least 7 days			
				Cuff: NR			
*Kido et al. (2015)*	M15	Habitually Active Healthy Males	24.0 ± 1.0	Thigh (bilateral)—proximal	Cycling	Ramp incremental exercise test:	No significant difference in VO_2_ kinetics in either condition. HHb amplitude and MRT significantly smaller in IPC during moderate-intensities. No significant difference in BLa in either condition
				>300 mmHg		30 W for 3-mins. 30 W⋅min^−1^ increments	
				3 × 5-mins		Work-to-work test:	
				Control: No cuff inflation		30 W for 3-mins. 90% GET for 4-mins (moderate domain). Severe domain - 70% difference in GET and VO_2peak_	
				1 IPC and 1 control session, separated by at least 7 days			
				Cuff: NR			
*Kilding, Sequeira, and Wood (2018)*	M8	Well-Trained Male Cyclists	27.0 ± 7.0	Thigh (bilateral)—proximal	Cycling	Ramp cycling test to exhaustion:	NC in VO_2peak_, VT or economy. NC in VO_2_ kinetics at moderate intensities. ↓ end VO_2_ and slow component at heavy intensities after IPC.
				200 mmHg		50 W for 3-mins. 20 W⋅min^−1^ increments	
				4 × 5-mins		Square wave protocol:	
				Sham: 50 mmHg		50 W for 6-mins at 80% VT.	
				3 IPC and 3 sham sessions (each before an exercise test) separated by 48–72 h		4-km TT	
				Cuff Width: 13” (FlexiPort Thigh 13′′ cuff, 40–55 cm, Welch Allyn, NY, USA)			
*Paradis-Deschenes et al. (2020)*	20	Endurance Trained Participants	IPC: 31.5 ± 3.0 Sham: 28.1 ± 2.5	Thigh (bilateral)—proximal	SIT Training/Cycling	Pre-post-testing:	NC in VO_2peak_
				220 mmHg		30s Wingate	↑ ΔHHb and ΔTHb after IPC in TT.
				3 × 5-mins		5-km TT	↑ ΔTSI after PLA from pre- and mid-testing to post-testing. ↑ ΔTSI after IPC in 5-km TT from mid-to-post testing
				Placebo: 20 mmHg		Maximal incremental step test:	NC ΔHHb and ΔTHb after the maximal test
				8 IPC or placebo sessions over 4-week, separated by a minimum of 2 days		Start: 100 W for 5-mins. 30 W⋅min^−1^ increments	↓ VEGF-α in both conditions. NC in HIF-1α
				Cuff Width: 21 cm (WelchAllyn, Skaneateles Falls, NY, USA)		4-week SIT training (2x per week)	↑ Fasting glucose after placebo
*Paradis-Descheneset al. (2016)*	M10	Strength-Trained Males	25.0 ± 4.0	Thigh (unilateral) - proximal	Knee Extension	5 sets—5 maximum knee extensions at 20^◦^⋅s^-1^ across 60^◦^	No meaningful change in ΔReoxy in IPC compared to sham. ↑ THb after IPC. ↑ ΔHHb_average_ and ΔHHb_peak_ after IPC.
				200 mmHg			
				3 ×5-mins			
				Sham: 20 mmHg			
				1 IPC and 1 sham session, separated by 3–7 days			
				Cuff Width: 21 cm (WelchAllyn, Skaneateles Falls, NY, USA)			
*Paradis-Deschenes et al. (2017)*	M9; F8	Strength-Trained Males and Females	M: 25.0 ± 2.0 F: 22.0 ± 1.0	Thigh (unilateral) - proximal	Knee Extension	5 maximum knee extensions at 20^◦^⋅s^−1^ across 60^◦^	↑ THb in males and females after IPC at rest and recovery
				200 mmHg			NC in O_2_Hb
				3 × 5-mins			NC in ΔHHb_peak_ after IPC.
				Sham: 20 mmHg			↑ ΔHHb_average_ in males set 1
				1 IPC and 1 sham session, separated by 3–7 days			↓ ΔHHb_average_ in females set 3/4/5
				Cuff Width: 21 cm (WelchAllyn, Skaneateles Falls, NY, USA)			
*Peden et al. (2022)*	M8	Strength-Trained Males	23.0 ± 2.0	Thigh (bilateral)—proximal	Cycling	Incremental TTE test (measure VO_2peak_ and MAP) 2x TTE test at power output equivalent to ∼92% MAP	↓ mean TSI during occlusion in IPC.
				220 mmHg			NC in TSI during occlusion/reperfusion in sham
				4 × 5-mins			NC in HHb mean response time in either condition
				Sham: 20 mmHg			Mitochondrial respiration—IPC limited leak respiration
				1 IPC and 1 sham session, separated by at least 7 days			
				Cuff: NR			
*Sabino-Carvalho et al. (2017)*	M14; F4	Well-Trained Runners	M: 22.3 ± 0.9 F: 24.0 ± 2.5	Thigh (bilateral)—proximal	Treadmill Running	Continuous treadmill test: 8 km⋅hr^−1^ for 3-mins. 1 km⋅hr^−1^ increments every minute. Discontinuous treadmill test: 1 km⋅hr^−1^ lower than VT (from continuous test) for 6-mins. 2 km⋅hr^−1^ for 3-mins then 1 km⋅hr^−1^ increments every 3-mins to exhaustion. Recovery walk at 5 km⋅hr^−1^ for 7-mins followed by a supra-maximal test	NC in VO_2,_ VO_2max_ or Lactate Threshold
				200 mmHg			
				4 × 5-mins			
				Sham: Simulated using therapeutic ultrasound			
				Control: Lying down, no application			
				1 IPC, 1 sham and 1 control session, separated by 7 days			
				Cuff: 17.5 cm (customised)			
*Tanaka et al. (2016)*	M12	Healthy Males	22.0 ± 1.0	Thigh (unilateral)—proximal	Knee Extension	3x MVC trials Isometric knee extension at 20% of MVC to failure	No significant difference in HHb between IPC and control. Shorter time delay for HHb after IPC
				>300 mmHg			
				3 × 5-mins			
				Control: Cuff - no inflation			
				1 IPC and 1 control session, separated by at least 7 days			
				Cuff: NR			
*Ter Beek et al. (2022)*	M14	Healthy Males	24.9 ± 2.1	Thigh (bilateral)—proximal	Cycling	2x Maximal incremental cycling test: 105 W warm-up. 35 W increments every 2-mins	NC in VO_2max,_ TSI, BLa, muscle deoxygenation (HHb) or oxygenation. Lower RPE recorded at 210 W and 245 W
				250 mmHg			
				4 × 5-mins			
				Sham: 20 mmHg			
				1 IPC and 1 sham session, separated by 7 days			
				Cuff Width: NR (Tourniquet cuff; VBM, Sulz am Neckar, Germany)			

Abbreviations: BLa, blood lactate; GET, gas exchange threshold; HHb, deoxygenated haemoglobin; ΔHHb, change in deoxygenated haemoglobin; IPC, ischaemic preconditioning; MAP, maximal aerobic power; MPO, mean power output; MVC, maximum voluntary contraction; NR, not reported; O_2_Hb = oxygenated haemoglobin; ΔO_2_Hb = change in oxygenated haemoglobin; PPO, peak power output; THb, total haemoglobin; ΔTHb, change in total haemoglobin; RPE, rating of perceived exertion; TSI, tissue saturation index; ΔTSI, change in tissue saturation index; TT, time trial; TTE, time to exhaustion; VO_2_ = oxygen uptake; VO_2max_ = maximal oxygen uptake; VT, ventilatory threshold.

### 3.2 Overview of studies

Seventeen studies were accepted in the review. A total of 237 participants were included, with an average sample size of 14 ± 4 participants. The distribution of sexes was predominantly male (78%), with 184 males, 21 females and 32 participants were not defined in the studies. Two studies did not report the sex of the participants (Cocking, et al., 2018; Paradis-Deschênes et al., 2020). However, these studies provided sufficient detail on the participants characteristics and satisified the inclusion threshold in the quality assessment. Of the 17 studies, the interventions included 11 cycling protocols, 4 knee extension exercises and 2 treadmill running protocols.

### 3.3 Ischaemic preconditioning

Table 2 presents the data extracted from the accepted articles, presenting the IPC protocols and the associated responses. There are variations in the IPC occlusion, but a reasonably standardized approach was adopted across the studies. Of the 17 IPC studies, the occlusion pressure ranged from 170 to 300 mmHg (Average: 222 ± 34 mmHg), with 53% using a standardized pressure of 220 mmHg and 24% using 200 mmHg. Only one study by Huang et al. (2020) used an individualized pressure of 50 mmHg above the Systolic Blood Pressure (SBP), with the remainder using a standardized pressure for all participants. The reported cuff width varied from 13.5 cm to 13 inches (∼33 cm) influencing the distribution of these pressures with 8 studies failing to report details on the cuff width. The occlusion and reperfusion cycles were almost exclusively performed with 3 or 4 cycles (47%: 53%), with all studies using a 5-min occlusion protocol. An additional trial was conducted by Cocking et al. (2018), finding no additional benefit from the 8 × 5-min protocol. There was more variation in the sham or control groups included in these studies. A sham procedure typically involves applying the cuff for the same duration of the IPC protocol but with a lower pressure applied, designed to mimic the IPC procedure and make the participants believe it is effective in a placebo effect (O’Brien and Jacobs, 2022). Ten studies as shown in Table 2 included a sham (i.e., 20 mmHg) or control intervention group or crossover trial, resulting in a limited impact on blood flow. One study also included a sham with a lower pressure of 10 mmHg (Huang et al., 2020) and one at 50 mmHg (Kilding, Sequeira, and Wood, 2018). An alternative method was to apply the cuff, but no inflation was performed (*n* = 5) (De Groot et al., 2010; Clevidence et al., 2012; Kido et al., 2015; Tanaka et al., 2016; Sabino-Carvalho et al., 2017).

#### 3.3.1 Physiological responses

Five studies investigated the effects of IPC on VO_2max_, with four studies identifying no changes in VO_2max_ (Sabino-Carvalho et al., 2017; Kilding et al., 2018; Jeffries et al., 2019; Ter Beek et al., 2022). De Groot et al. (2010) was the only study finding a significant increase in VO_2max_ (*p* = .003) in the IPC condition following a single session of IPC, associated with a small effect size (*d =* 0.25, power = 0.10). Similar results were identified for VO_2peak_, where Cruz et al. (2015) evidenced a significant increase (*p =* .040) with a trivial effect size (*d =* 0.10, power = 0.04). Neither Paradis-Deschênes et al. (2020) and Griffin et al. (2018) observed any difference in VO_2peak_ in either condition both with trivial or small effects respectively (placebo: *d =* −0.15, IPC: *d =* −0.35; *d =* −0.1 respectively).

#### 3.3.2 Metabolic responses

The primary metabolite analyzed was blood lactate, recorded in ten studies, with no difference consistently reported in either IPC or sham conditions in seven of these studies, or in lactate threshold (Sabino-Carvalho et al., 2017). Jeffries et al. (2019) evidenced a significant increase (*p* < 0.001) in end blood lactate during the maximal test with no differences sub-maximally. Interestingly, Cocking et al. (2018) evidenced a non-significant increase (*p* = .06) in blood lactate during the 4 × 5-min method but observed a significant increase (*p* = .006) increase during the 8 × 5-min protocol compared to sham conditions.

Only two studies measured blood glucose with contrasting findings. Clevidence, Mowery and Kushnick (2012) found no significant difference in glucose between conditions, whereas Paradis-Deschênes et al. (2020) observed a higher fasting glucose in the placebo condition, corresponding to an increase in insulin levels in the IPC condition.

#### 3.3.3 Vascular responses

The PICO strategy incorporated vascular responses. However, no IPC studies accepted in this review fulfilled this outcome variable.

#### 3.3.4 Oxygenation

Oxygenation measures primarily incorporated a combination of oxygenated (O_2_Hb), deoxygenated (HHb) and total haemoglobin (THb) concentration in the tissue, and tissue saturation index (TSI) estimated using near-infrared spectroscopy (NIRS).

Studies performed by Huang et al. (2020), Paradis-Deschênes, Joanisse and Billaut (2017) and Ter Beek et al. (2022) produced no change in O_2_Hb between IPC and SHAM. Paradis-Deschênes, Joanisse and Billaut (2016) found no change in muscle reoxygenation rate after IPC (*d* = −0.10, power = 0.04).

Mixed results were identified for HHb as assessed in nine studies. Similar to O_2_Hb, there was no change reported during IPC or sham conditions by Huang et al. (2020) and Ter Beek et al. (2022), further supported by Tanaka et al. (2016). Other measures of HHb including the mean response time ( Mean Response Time=Time Delay+Time Constant ) (Kido et al., 2015) were significantly quicker (*p* < .05) during the IPC trials when performing moderate-intensity exercise with large effects (*d* = 3.45, power = 1.00) (Kido et al., 2015). Whereas Peden et al. (2022) found no difference in mean response time in either the IPC or sham conditions.

Paradis-Deschênes, Joanisse and Billaut (2016) identified an increase in ΔHHb_average_ and ΔHHb_peak_ following IPC compared to sham conditions. In a similar study investigating sex differences by Paradis-Deschênes, Joanisse and Billaut (2017), ΔHHb_average_ increased in males during set 1 of knee extensions, whereas ΔHHb_average_ decreased in females during sets 3–5 with no change in ΔHHb_peak_. However, there was no difference and trivial effects in the baseline levels of HHb in both males (*d* = −0.11, power = 0.04) and females (*d* = 0.08, power = 0.04) when comparing sham and IPC. Different protocols may also influence ΔHHb, with increases after a 5-km cycling time trial, but no changes during a maximal incremental cycling test (Paradis-Deschênes et al., 2020). In contrast, Jeffries et al. (2019) found a decrease during sub-maximal cycling performed at 70% (*p* = .007), 80% (*p* = .010) and 90% (*p* = .017) of the ventilatory threshold during the IPC condition, with small (*d =* 0.47, power = 0.19), medium (*d =* 0.67, power = 0.32) and small (*d =* 0.42, power = 0.16) effect sizes respectively.

Total haemoglobin concentration in the tissue was assessed in five studies. Despite changes in HHb, there was no overall change in THb across the different intensities when performing sub-maximal cycling at 70%, 80%, and 90% of the ventilatory threshold following 7 days of IPC (post-testing sham vs IPC: *d*_mean_ = 0.24) (Jeffries et al., 2019). Similar results to HHb were replicated for THb with increases in ΔTHb after a 5-km time trial and no change after the maximal test following a 4-week intervention (Paradis-Deschênes et al., 2020). Huang et al. (2020), Paradis-Deschênes, Joanisse and Billaut (2016) and Paradis-Deschênes, Joanisse and Billaut (2017) all reported increases in THb after a single session of IPC, with the latter occurring in both males and females. The male group resulted in a trivial effect size (*d* = −0.07, power = 0.04) whereas the female group had a medium effect size (*d* = 0.68, power = 0.27).

Tissue saturation index was only reported in four studies. Peden et al. (2022) and Griffin et al. (2018) produced large decreases in TSI only during the occlusion phase of IPC, with no effects during sham conditions (*d* = 5.08, power = 1; *d* = 18.98, power = 1 respectively). However, overall, Griffin et al. (2018) and Ter Beek et al. (2022) found no overall change in TSI with trivial effects (*d* = −0.16, power = 0.06; *d* = 0.12, power = 0.5 respectively). To the contrary, Paradis-Deschênes et al. (2020) evidenced decreases in absolute TSI, represented by increases in ΔTSI, in both IPC and placebo (20 mmHg) conditions. Notably, IPC increased ΔTSI only between mid-to-post training while maintaining stable results for the duration of the time trial.

#### 3.3.5 Hypoxia blood markers

##### 3.3.5.1 Vascular endothelial growth factor (VEGF)

There were no studies accepted which fulfilled the outcome for gene expression. However, Paradis-Deschênes et al. (2020) has reported blood markers which are indicative of vasodilation and angiogenesis. Paradis-Deschênes et al. (2020) recorded decreases in VEGF with a large effect size (placebo: *d =* 1.16, power = 0.69; IPC: *d* = 0.96, power = 0.61).

##### 3.3.5.2 Hypoxia induced factor 1α (HIF-1α)

Paradis-Deschênes et al. (2020) also found no changes in HIF-1α in both IPC and placebo conditions with trivial effects (placebo: *d =* −0.14, power = 0.05; IPC: *d* = −0.16, power = 0.06).

## 4 Discussion

### 4.1 Physiological responses

De Groot et al. (2010) was one of the few studies to identify an improvement in VO_2max_, which may be attributed to the combination of study design and blinding. Four studies identified no changes in VO_2max_ (Sabino-Carvalho et al., 2017; Kilding, Sequeira, and Wood, 2018; Jeffries et al., 2019; Ter Beek et al., 2022). These were all performed with a randomized crossover study design, whereas De Groot et al. (2010) designated the IPC application in a counterbalanced order. For the determination of VO_2max_, these five studies used the highest average 30-s, except for Sabino-Carvalho et al. (2017) who adopted 20-s and performed a verification follow up test. However, there were also differences in the training status, protocols and exercise modalities adopted for these different studies which is likely to affect the determination of VO_2max_. Despite these five studies applying similar IPC manoeuvres, De Groot, et al. (2010) was the only study evidencing improvements. This improvement could be influenced by the lack of randomization, blinding or sham condition present in this study, contributing to an expectancy or placebo effect. This does not negate participants having prior awareness of the potential benefits of IPC, particularly as this study was performed in a trained population. These improvements in VO_2max_, could be concurrent with the significant increase in power output (De Groot et al., 2010), particularly supporting the idea that there are other factors such as training status and exercise modality resulting in the increase in VO_2max_.

Further conflicting findings occurred for VO_2peak_, with Cruz et al. (2015) identifying a significant increase compared to Paradis-Deschênes et al. (2020) and Griffin et al. (2018). Despite these differences, these three studies all resulted in a trivial or small effect size limiting the meaningfulness of the significant result for Cruz et al. (2015). The cumulative effect of IPC over a 4-weeks intervention had no impact on VO_2peak_ (Paradis-Deschênes et al., 2020). It is important to consider that the use of VO_2peak_ over VO_2max_ in these studies may imply that there was no control over the VO_2_ analysis and may not represent a true maximum value (Poole and Jones, 2017). It is difficult to attribute the unchanged VO_2peak_ to the length of the intervention or whether this was affected by other potential flaws, especially with the lack of certainty regarding mechanisms for IPC. For example, this could be attributed to the lower number of IPC cycles (3 cycles) compared to the other studies (4 cycles) resulting in a reduced occlusion time which may be an ineffective training stimulus in a trained population. However, improvements were evidenced for VO_2max_ with the same number of occlusion cycles in the study by De Groot et al. (2010). Despite this, this study promoted a high-quality rating. Irrespective of the outcomes, Cruz et al. (2015) and Paradis-Deschênes et al. (2020) benefitted from a reliability assessment, which are some of the few studies in the entirety of this review to conduct a reliability assessment, a fundamental flaw acknowledged in the limitations of this review.

### 4.2 Lactate responses

The effects of IPC on blood lactate have been investigated from two perspectives, where studies such as Jeffries et al. (2019) and Kaur et al. (2017) have identified significant increases from resting baseline samples to post-exercise. These findings are reasonable to expect due to increases above the lactate threshold resulting in an accumulation of blood lactate (Kaur et al., 2017). Cocking et al. (2018) also identified increases which were all performed at a pressure of 220 mmHg with a 13.5 cm cuff, with a lower blood lactate during the extended 8 × 5-minutes protocol. However, this remained significant during the 4 × 5-minutes protocol, consistent with the overall conclusion that there is no additional benefit from the larger 8 × 5-minutes protocol. Ultimately, when the majority of studies compare the effects on blood lactate in IPC compared to placebo or sham conditions performed at either the finger or earlobe, there are no differences between conditions (De Groot et al., 2010; Clevidence et al., 2012; Cruz et al., 2015; Kido et al., 2015; Kaur et al., 2017; Sabino-Carvalho et al., 2017; Griffin et al., 2018; Ter Beek et al., 2022). While these changes in blood lactate will be affected by individual variations and dependent on the exercise protocols adopted in each study, with maximal work potentially eliciting a higher measurement, there is the potential that IPC may have a minimal contribution on lactate metabolism (Clevidence et al., 2012; Sabino-Carvalho et al., 2017).

### 4.3 Vascular responses

Despite including vascular responses in the search strategy, this review did not identify any vascular outcomes from the accepted articles. The proposed effects of IPC impact the vascular mechanisms, such as the increase NO production resulting in vasodilation and an increase in shear stress (Gu et al., 2021). This highlights a gap in the literature for the assessment of vascular responses to IPC. Furthermore, there are contraindications and safety considerations for the use of BFR and IPC such as varicose veins and cardiovascular disease (Brandner et al., 2018). Therefore, it is important to consider the participants’ vascular health status primarily to assess for peripheral arterial disease, by using the Ankle Brachial Index (ABI) as part of the recruitment criteria (Loenneke et al., 2012). It has also been recommended to utilize lower occlusion pressures that still produce the desired effects, rather than an absolute pressure for all participants which may not achieve a complete occlusion (McEwen et al., 2019). This can be achieved using individualized arterial occlusions pressures (AOP) (Brandner et al., 2018). However, only one study (Huang et al., 2020) used an individualized pressure with 16/17 using a fixed standardised pressure. The use of individualized pressures is not only a safer alternative, but provides less variability in the responses and reduces the risk of injury and adverse side effects (McEwen et al., 2019).

### 4.4 Haemoglobin responses

#### 4.4.1 Oxy-, deoxy- and total-haemoglobin

Studies performed by Huang et al. (2020), Paradis-Deschênes et al. (2016), Paradis-Deschênes et al. (2017) and Ter Beek et al. (2022) all concluded that IPC provided no significant effect on O_2_Hb. The study of Paradis-Deschênes, Joanisse and Billaut (2017) was also the only one of the four IPC studies to include female participants, benefiting from an even distribution of sexes. The resultant performance effects of this study identified increased force in males, compared to females, consistent with Paradis-Deschênes, Joanisse and Billaut (2016), indicative that IPC could be more effective in male populations. However, the mechanisms remain unclear. Critically, the lack of research investigating female participants highlights a key limitation and raises a caution to not draw false inferences due to the potential disparities of IPC in males compared to females. Previous research has also indicated that there may be differences in the AOP of males and females (Jessee et al., 2016). However, the same standardized pressure was used in both male and females for this study (Paradis-Deschênes et al., 2017). It appears that differences in occlusion pressure ranging from 170 mmHg up to 300 mmHg were not effective in generating any differences in the overall results. The variation in the application does not seem to be associated with an optimal or gold standard method at present, requiring further clarification in the future. There are further implications that a higher pressure may not elicit any additional beneficial responses, highlighting a lower pressure may be more appropriate from a safety perspective to avoid using higher pressures unnecessarily. This could be partly attributed to variations in the method of IPC application or potential weaknesses in the studies studies such as a lack of females and potential issues with the pressure applied. However, there is a likelihood that the lack of change in O_2_Hb suggests that the greatest change in THb is ultimately influenced by HHb.

Huang et al. (2020) was the only IPC study to use individualized occlusion pressures which McEwen et al. (2019) have suggested to be vital for occlusions, although a standardized pressure of 50 mmHg above an individual’s Systolic Blood Pressure (SBP) was adopted. A key issue with this method adopted by Huang et al. (2020) was the body position in which the occlusion pressure was determined, recorded while seated, but the IPC was applied supine, which has been found to have significant effects on occlusion pressure (Karanasios et al., 2022). Huang et al. (2020) has attributed this rationale to differences in arm and leg pressures, when SBP is measured in the arm compared to higher pressures present in lower limbs, similar to the higher pressures while supine compared to seated. Furthermore, the pressure was not confirmed as a venous or arterial occlusion during the IPC manoeuvre, but it is possible that the average pressure of 170 mmHg, calculated from SBP plus 50 mmHg, would be insufficient to create a complete arterial occlusion in the femoral artery. If this pressure was insufficient, this could explain the lack of change in O_2_Hb and HHb. Notably, this was the only IPC study to perform the occlusion at the anterior midline of the thigh, which may account for the lower pressure compared to the proximal thigh.

Exercise testing is typically performed in close succession after the utilization of IPC, whereas Paradis-Deschênes et al. (2020) was the only accepted IPC study utilizing a 4-week intervention for the repeated exposure to IPC, where the cumulative effects of training and IPC resulted in an increased change in HHb in cycling time trial performance, but not during maximal cycling. A similar trend was evidenced with Kido et al. (2015) with a greater effect on HHb in the moderate exercise domain, but not during the severe domain (domains defined in Table 2). The severe domain did offer other improvements such as an increased time to exhaustion despite not influencing HHb. This could suggest that the intensity and physiological load of the exercise may also be an influencing factor especially as the same IPC manoeuvre was applied in both domains. Contrary to this, Jeffries et al. (2019) found a decrease in the change in HHb during sub-maximal cycling, referring to the cumulative effect of IPC as opposed to one-off sessions that may be reliant on timing the window of protection. The first window of protection occurs approximately 2–3-h following the application of IPC, with a second window in the 24–72-h following application resulting in cardiovascular and vascular protective effects (Riksen et al., 2004). Despite other studies recruiting untrained healthy individuals, Haung et al. (2020) was the only study to modify the warm-up and IPC interventions to a non-athletic healthy population group. Although this has benefits from a safety perspective, combined with the lower occlusion pressure, the training stimulus may have been insufficient to create an arterial occlusion or training benefits hence the lack of change in HHb. Despite using different interventions and significantly different occlusion pressures, Ter Beek et al. (2022) also found no significant difference in a HHb at 250 mmHg, similar to Tanaka et al. (2016) at 300 mHg suggesting no additional benefit at higher occlusions. These differences are unlikely to be attributed solely to the method of IPC application as varying occlusion pressures from 220 mmHg to 300 mmHg resulted in some change in HHb (Paradis-Deschênes et al., 2020; Kido et al., 2015 respectively) compared to the latter studies adopting 250 mmHg to 300 mmHg. While these pressures would be influenced by the cuff width distributing the pressure, three of these studies failed to report the cuff width.

Contrary to this, Kido et al. (2015) and Tanaka et al. (2016) identified accelerated deoxygenation dynamics during moderate intensity exercise during the application of IPC also performed at a pressure of 300 mmHg. Although the interventions were different, both used endurance-based methods and applied the same IPC pressure (300 mmHg) However, a notable difference was the implementation of a control group without a sham pressure, where a cuff was applied without inflation. As highlighted by Peden et al. (2022), it is inherently difficult to blind participants due to the obvious pressure differences between conditions, often resulting in a potential for bias. With these studies using the greatest occlusion pressure out of the 17 IPC studies, there could be an enhanced expectancies effect during the control conditions. However, it is difficult to attribute if differences should be accounted for by the occlusion protocol, or other control factors such as placebos which can be administered using different techniques. Indeed, Peden et al. (2022) attempted to blind participants from feedback and deceived the participants through a 20 mmHg sham but not informing them of the reasoning for an occlusion to limit a placebo effect. In comparison, Paradis-Deschênes et al. (2016), Paradis-Deschênes et al. (2017) also adopted a 20 mmHg sham, but the participants were informed that the purpose was to investigate cuff pressure. These different explanations were both designed to limit a placebo effect from the noticeable difference between sham and IPC trials. Unlike Peden et al. (2022), these studies resulted in increases in ΔHHb_average,_ with Paradis-Deschênes et al. (2017), identifying different but trivial effects in both males and females.

The resultant effects of HHb produced similar effects on THb, indicating changes in HHb was the driving component influencing THb due to the lack of change in O_2_Hb. THb increases blood flow as a known effect of IPC (Paradis-Deschênes et al., 2017). In studies performed by Paradis-Deschênes et al. (2020), Paradis-Deschênes, Joanisse and Billaut (2016) and Paradis-Deschênes, Joanisse and Billaut (2017), these all reported some increases in THb indicative of an increase in HHb, but no change in O_2_Hb. Contrary to the suggestion that IPC may be more effective in males, the effect of THb was trivial in males (*d* = −0.07), with a medium effect in females (*d* = 0.68), highlighting the necessity for future research to include and differentiate female participants. While Huang et al. (2020) also identified increases in the resting THb, blood flow, and therefore haemoglobin, was not continuously measured throughout the occlusion, making it difficult to accurately assess which component influenced the increase. Considering that Huang et al. (2020) found no changes in O_2_Hb or HHb, the increased THb could be indicative of arterial blood to the limb evidencing a venous occlusion rather than an arterial occlusion, further highlighted by the lowest occlusion pressure. In contrast, Jeffries et al. (2019) benefited from measuring the limb occlusion pressure (LOP) in the leg to ensure an arterial occlusion has occurred resulting in no change in THb but proceeded to use the standardized 220 mmHg approach as opposed to the recorded LOP. Notably, there was no change in THb at different workload intensities, nor following a 7 day repeated IPC intervention compared to the acute interventions applying a single sham and IPC condition separated by 3–7 days in the studies by Paradis-Deschênes et al. (2016), Paradis-Deschênes et al. (2017) and Huang et al. (2020).

#### 4.4.2 Tissue saturation index

Peden et al. (2022) and Griffin et al. (2018) observed significant decreases with a large effect size and good power in TSI during the four occlusion phases, as expected due to the reduction in oxygenated blood to the effected limb during an arterial occlusion. Critically, the overall TSI used as an indicative measure of muscle oxygenation did not change resulting in trivial effects at the point of exhaustion (Griffin et al., 2018; Ter Beek et al., 2022), consistent with findings for O_2_Hb. Although unlikely to have a significant effect, TSI was averaged over different time periods within these studies, with Ter Beek et al. (2022) using the shortest 15-s averaging which is important to consider for a valid comparison of these measures. Paradis-Deschênes et al. (2020) recorded increases in both mean and peak ΔTSI, indicative of a decrease in absolute TSI values, assessed over the time trial duration, as opposed to during individual occlusions. Both IPC and placebo conditions result in increases in ΔTSI during the 5-km time trial, highlighting a potential training effect of the 4-week intervention. More specifically, the delayed effects of IPC could be attributed to the IPC condition only increasing ΔTSI from mid-to-post training. Consistently across these studies, the participants were not informed of the true nature of the study to limit placebo effects. These four studies have identified similar results for TSI, and despite differences in the protocols and tests used, the studies all performed maximal cycling interventions with similar occlusions and population groups. Considerations should be made that these results may only be applicable to cycling interventions, and conclusions should not be drawn for other exercise interventions to avoid drawing false inferences.

### 4.5 Hypoxia blood markers

The ischaemia present during the occlusion creates a localized hypoxic environment below the occlusion (Li et al., 2022), leading to the expression of HIF-1α (Hypoxia-Induced Factor 1-alpha) as a protective factor and encoding for VEGF (Vascular Endothelial Growth Factor). This can lead to the development of angiogenesis during the application of IPC in repeated interventions (Chen et al., 2015), as opposed to the single sessions of IPC commonly identified in this review. Despite the expression of genetic components relating VEGF and HIF-1α to angiogenesis, there were no studies accepted which measured the gene expression. Paradis-Deschênes et al. (2020) was the only accepted IPC study to record the expression of VEGF and HIF-1α measured through blood markers indicative of hypoxia and angiogenesis. The reported decrease in VEGF and lack of change in HIF-1α counters the expected result. However, Paradis-Deschênes et al. (2020) has attributed this to limitations in the methodology by only taking one measurement, whereas different time periods or repeated measures of the blood markers may improve the reliability of the results (Taylor et al., 2016). Furthermore, there are suggestions that the blood measurements 2-day following the final intervention may miss the window of protection for IPC (Paradis-Deschênes et al., 2020). However, this study focused on reliability and keeping participants stable between visits, incorporating the use of training logbooks and replicating training routines throughout the study to limit the influence of external variables. There are implications that by keeping other variables stable promoting good reliability, and improvements in some measures of oxygenation, that the long-term effects may not be achievable in a 4-week intervention.

## 5 Limitations

The review identified considerable diversity in the outcomes and heterogeneity of the available data, for examples, the units in which the NIRS data were recorded and reported making it difficult to compare these findings. The variation in the results obtained could be attributed to the breadth of the review question and search strategy, particularly in relation to study design. The variability highlights a limitation in this review due to the variance in the type of exercise and protocols utilized, making it difficult to compare as there was no standardized approach. Furthermore, at least 78% of the population in the studies utilised male participants (184 out of 237 participants) identifying a significant gap in the literature and making it difficult to draw inferences on the effect of biological sex. There were no female only studies, with only 9% of the population in the accepted studies including female participants (21 out of 237 participants). Paradis-Deschênes, Joanisse and Billaut (2017) suggests that this could also be attributable to IPC being more effective in males than females. However, additional research would be required to support this finding and is necessary to incorporate into future research.

The influence of cuff width has been highlighted in the literature (Loenneke et al., 2012), with a wider cuff requiring a lower pressure due to the greater distribution of pressure. Jessee et al. (2016) suggested that an individual’s limb circumference is one of the main contributing factors influencing AOP when applying different cuff widths, highlighting the importance of reporting cuff width. However, many studies have failed to report the cuff width with only 9 out of 17 articles accepted reporting the width as shown in Table 2. This is an issue that has also been raised by Loenneke et al. (2012) in the wider literature.

In the absence of an arterial occlusion, sham or control groups (typically 20 mmHg or no occlusion) typically adopted a low occlusion pressure, designed to have no influence but created a placebo effect. Peden et al. (2022) highlights the fundamental issue of blinding participants due to the obvious difference in pressure. This raises the issue of placebo and nocebo effects, where some protocols opted to deceive participants by telling them an alternative reason or alternatively, applying a cuff with no inflation. These are not limitations of the review *per se* but should be accounted for due to the inherent difficulties associated with blinding during occlusions.

## 6 Conclusion

The aim of this systematic literature review was to investigate differences in the methods adopted in ischaemic preconditioning, and the associated physiological responses. There were mixed results across all measures assessed including the oxygenation, lactate and vascular responses, creating the opportunity for further research to clarify the responses and the mechanisms associated with these. Consistently, there have been conflicting findings for IPC. While there are variations in the method of application, the overall application is 3 or 4 cycles, with an average occlusion of ≈220 mmHg. The majority of these studies has applied the occlusion at proximal portion of the thigh, with gaps in the research investigating lower limb occlusion below the thigh with no additional benefit from higher occlusion pressures or more occlusion cycles. This is also associated with other gaps with many studies not assessing the reliability of the measures used. While there are some consistencies in the methods used, there has been little justification for these and primarily established by previous research. Further to this, there are gaps in the reporting of the methodologies specifically details regarding the cuff width for the application of an occlusion. Despite the lack of consensus leading to a fully established gold standard protocol, the results indicate agreement in specific aspects of the protocols including the location of the occlusion, typical pressures used and limited benefits of higher pressures during the occlusion phases. Further research is required to refine the protocols and associated responses which in turn will facilitate a stronger evidence base for research and practice.

## Data Availability

The original contributions presented in the study are included in the article/Supplementary material, further inquiries can be directed to the corresponding author.

## Abbreviations

HHb, deoxygenated haemoglobin; IPC, ischaemic preconditioning; O_2_Hb, oxygenated haemoglobin; THb, total haemoglobin; TSI, tissue saturation index.
